# Assessing the reliability and validity of the Italian EQ-5D-5 L in multiple sclerosis: a cross-sectional study

**DOI:** 10.1186/s41687-026-01077-2

**Published:** 2026-04-30

**Authors:** Giovanni Sellitto, Elisa Simonazzi, Francescaroberta Panuccio, Andrea Marini Padovani, Emanuele Amadio, Rachele Simeon, Anna Berardi, Giovanni Galeoto, Ilaria Ruotolo

**Affiliations:** 1 Department of Human Neurosciences, Sapienza University of Rome, Rome, Italy; 2 Department of Public Health and Infectious Disease, Sapienza University of Rome, Rome, Italy; 3 Sapienza University of Rome, Rome, Italy; 4 Department of Neuroscience, Rehabilitation, Ophthalmology, Genetics and Maternal Child Health (DINOGMI), University of Genoa, Genoa, Italy; 5 Istituto Neurologico Mediterraneo, Pozzilli, Italy

**Keywords:** EQ-5D-5L, Health-Related Quality of Life (HRQoL), Multiple Sclerosis, Patient-Reported Outcome Measures (PROMs), Psychometric properties

## Abstract

**Background:**

Multiple Sclerosis (MS) significantly affects physical, cognitive, and psychosocial functioning, leading to impaired Health-Related Quality of Life (HRQoL). The EQ-5D-5 L is a widely used generic patient-reported outcome measure (PROM) to assess HRQoL across health conditions. However, its psychometric properties have not been fully established in Italian people with MS (PwMS). This study aimed to evaluate the reliability and validity of the Italian EQ-5D-5 L in PwMS, supporting its applicability in clinical and research contexts.

**Methodology:**

A cross-sectional psychometric validation study was conducted in accordance with COSMIN guidelines. Participants were recruited from an Italian tertiary MS center based on the following inclusion criteria: age ≥ 18 years, confirmed MS diagnosis according to the 2017 McDonald criteria, fluency in Italian, and ability to provide informed consent and independently complete the questionnaire. Eligible participants completed the Italian EQ-5D-5 L and the MSQOL-54 in person. Test-retest reliability was assessed via Intraclass Correlation Coefficients (ICC) after a 48-hour interval in clinically stable participants, construct validity through Pearson’s correlations with MSQOL-54 domains, and known-groups validity by ANOVA and t-tests across disability levels (EDSS), employment status, and physiotherapy engagement.

**Results:**

Sixty-nine PwMS (55% female; mean age 49 ± 13 years) were included. The EQ-5D-5 L showed high test–retest reliability (ICC range = 0.81–0.95). Strong correlations supported construct validity, particularly between the EQ-Index and the Physical Health Composite Score (*r* = 0.76, *p* < 0.001) and between the EQ-VAS and the Overall Quality of Life item (*r* = 0.71, *p* < 0.001). Known-groups validity was confirmed, with higher HRQoL scores among participants with mild disability (EDSS 0–3.5) and those employed (both *p* < 0.001).

**Conclusions:**

The Italian EQ-5D-5 L showed satisfactory reliability and construct validity in PwMS, supporting its use as a generic measure of HRQoL in clinical and research settings. The relatively small sample size, particularly in the test–retest subgroup, should be considered when interpreting these findings. Additional measurement properties, including content validity, were not specifically evaluated in the present study. Complementary use with MS-specific instruments remains advisable to capture domains such as fatigue and cognitive function.

## Background

Multiple Sclerosis (MS) is a chronic and progressive immune-mediated demyelinating disorder of the central nervous system (CNS), which affects over 2.8 million individuals worldwide [1]. Europe reports some of the highest prevalence rates globally, with Italy showing approximately 176 cases per 100,000 inhabitants and a female-to-male ratio of 2:1 [2]. The disease most commonly manifests between the ages of 20 and 40 and is characterized by diverse clinical phenotypes, including Relapsing-Remitting MS (RRMS), Secondary Progressive MS (SPMS), and Primary Progressive MS (PPMS), each contributing uniquely to long-term disability progression and patient health outcomes [3].

The burden of MS extends beyond physical impairments, affecting people’s psychological, social, and economic well-being [4]. Health-Related Quality of Life (HRQoL) has emerged as a critical outcome in managing chronic diseases like MS, given its ability to reflect the broader impacts of illness on daily functioning and overall life satisfaction. HRQoL measures capture people’s perspectives on their health status, making them essential for tailoring interventions and evaluating treatment effectiveness [5–8].

Health-related quality of life in people with multiple sclerosis (PwMS) is influenced by a combination of physical disability, fatigue, cognitive impairment, emotional distress, and social factors [5, 9–12]. These determinants highlight the need for multidimensional outcome measures capable of capturing both physical and psychosocial aspects of disease impact. Previous studies have shown that disability severity, fatigue, and depressive symptoms are among the strongest predictors of reduced HRQoL across both generic and MS-specific instruments [5, 9, 13]. These findings support the use of patient-reported outcome measures that can adequately reflect the complex and heterogeneous impact of MS on daily functioning and well-being.

Patient-reported outcome measures (PROMs) are central to the assessment of HRQoL in chronic neurological conditions, as they capture the individual’s perspective on the impact of disease on daily functioning and well-being [14, 15]. In MS, several condition-specific instruments have been developed to assess HRQoL and disease impact more directly, including the Multiple Sclerosis Quality of Life-54 (MSQOL-54) [16], the Functional Assessment of Multiple Sclerosis (FAMS) [17], the Multiple Sclerosis Impact Scale (MSIS-29) [18], and the Multiple Sclerosis International Quality of Life questionnaire (MusiQoL) [19]. These measures were designed to reflect domains that are especially relevant in MS, such as fatigue, cognitive difficulties, emotional burden, and the broader consequences of the disease on everyday life [9].

However, disease-specific instruments do not fully replace the role of generic measures. The EQ-5D-5 L, developed by the EuroQol Group, is a widely used generic preference-based PROM that assesses five core dimensions of health—mobility, self-care, usual activities, pain/discomfort, and anxiety/depression—alongside a visual analogue scale (EQ-VAS) for self-rated overall health status [20–23] Its main advantage is that it allows comparisons across diseases, populations, and healthcare settings, while also generating utility values that are particularly relevant for health services research and economic evaluation [24, 25].

In the MS field, this generic perspective remains important for at least two reasons. First, it provides a standardized metric for comparing the burden of MS with that of other chronic conditions and for supporting resource-allocation and policy-oriented evaluations. Second, its brevity and feasibility make it suitable for routine use in both clinical practice and observational research. Because the EQ-5D-5 L is not disease-specific, it may be less sensitive to domains that are particularly relevant in MS, such as fatigue and cognitive dysfunction. These limitations should be considered when interpreting HRQoL results obtained with generic instruments, especially in conditions characterized by complex and multidimensional symptom profiles.

Therefore, the relevance of the EQ-5D-5 L in PwMS lies not in replacing MS-specific instruments, but in complementing them with a concise, standardized, and preference-based measure of overall HRQoL. Despite its widespread international use, its psychometric performance has not yet been specifically established in Italian people with MS. For this reason, the aim of the present study was to evaluate the reliability and validity of the Italian EQ-5D-5 L in PwMS and to examine its ability to discriminate across clinically distinct subgroups defined by disability severity.

## Methods

### Study design

This study employed a cross-sectional psychometric validation design to evaluate the measurement properties of the Italian version of the EQ-5D-5 L in people with Multiple Sclerosis (PwMS). A cross-sectional design was considered appropriate, as the study aimed to assess reliability and validity at a single time point rather than responsiveness to change.

The study was conducted in accordance with the COnsensus-based Standards for the selection of health status Measurement INstruments (COSMIN) guidelines to ensure a rigorous evaluation of validity and reliability indicators [26]. All procedures complied with the ethical standards of the institutional and national research committees and with the Declaration of Helsinki (1975, revised in 2008). The protocol was approved by the Comitato Etico Territoriale Lazio Area 1 (regional determination no. G01659 of 10/02/2023; approval date: 06/11/2023; Protocol No. 0969/2023). All participants provided written informed consent prior to enrolment.

The study was not registered in a clinical trial registry, as it involved a non-interventional psychometric validation protocol.

### Participant recruitment and eligibility criteria

Participants were consecutively recruited between November 2023 and October 2024 from individuals attending the Multiple Sclerosis Center of Policlinico Umberto I, Sapienza University of Rome. A non-probabilistic convenience sampling strategy was adopted to ensure feasibility in a real-world clinical setting while allowing inclusion of participants with heterogeneous clinical characteristics.

Inclusion criteria included:

- Age ≥ 18 years.
- Confirmed diagnosis of MS based on 2017 McDonald criteria [27].
- Proficiency in the Italian language.
- Ability to provide informed consent and autonomously complete the questionnaire.

Exclusion criteria were:

- Severe cognitive impairments or psychiatric/neurological comorbidities (e.g., stroke, major depressive disorder) that could interfere with the reliable completion of the questionnaire.
- Clinical relapse or corticosteroid treatment in the previous 30 days.

### Data collection procedures

Each participant independently completed the self-administered Italian versions of the EQ-5D-5 L and MSQOL-54 questionnaires during face-to-face sessions conducted in a quiet consultation room, with assistance available if required. When required, assistance was limited to procedural clarification (e.g., instructions on questionnaire completion) and did not involve interpretation of item content or guidance in response selection.

Demographic and clinical data were obtained through structured interviews and consultation of participants’ medical records. Variables were grouped into continuous, dichotomous, and categorical categories to facilitate analysis and interpretation. Categorization of selected variables was performed to allow clinically interpretable subgroup comparisons and to avoid sparse data in stratified analyses. Age was recorded as a continuous variable. In contrast, gender and employment status were dichotomized into binary categories.

Educational attainment was categorized into six levels: elementary school, middle school, high school diploma, bachelor’s degree, master’s degree, and PhD. Time since MS diagnosis was categorized into five intervals: less than 10 years, 10–14 years, 15–19 years, 20–29 years, and more than 30 years. Disability levels, assessed using the EDSS, were grouped into three categories: mild (EDSS 0–3.5), moderate (EDSS 4–6), and severe (EDSS 6.5–9).

Engagement in physiotherapy and Body Mass Index (BMI) classification (underweight, normal weight, overweight) were also recorded to allow a broader exploratory analysis of quality-of-life variations. However, these variables were not considered as core indicators of sample representativeness.

A subsample of participants repeated the EQ-5D-5 L questionnaire after 48 h to evaluate test-retest reliability. This short interval was selected to minimize recall bias while reducing the likelihood of change in health status. Inclusion in the test-retest group required clinical stability, operationally defined as absence of acute relapses, significant symptom fluctuations, or recent changes in disease-modifying treatments during the interval. Similar short intervals have been used in previous psychometric studies in neurological populations to minimize recall bias while ensuring clinical stability.

### Outcome measures

The EQ-5D-5 L is a widely used generic measure of HRQoL and consists of two main sections:

1. *Descriptive System*: This section assesses five dimensions of health—mobility, self-care, usual activities, pain/discomfort, and anxiety/depression. Each dimension is rated on a five-level severity scale, ranging from no problems to extreme problems. The descriptive system generates a five-digit health profile code for each participant, representing different health states. In total, the EQ-5D-5 L can describe 3,125 unique health states. These health states can be converted into a single summary index value using country-specific value sets, which reflect societal preferences for different health conditions. For this study, the Italian value set was used to derive the EQ-5D-5 L index scores [28, 29].
2. *Visual Analogue Scale (VAS)*: The VAS allows participants to rate their overall health on a continuous scale from 0 to 100, where 0 represents the “worst imaginable health” and 100 indicates the “best imaginable health.” This component provides an overall self-assessment of health status, offering insights into participants’ perceived quality of life that may not be fully captured by the descriptive system.

The EQ-5D-5 L was selected because of its widespread international use, availability of an Italian value set, brevity, and relevance for cross-condition comparisons and utility-based evaluations.

The MSQOL-54, a disease-specific HRQoL measure, was used as a comparator to assess construct validity [16]. Derived from the Short Form (SF-36) Health Survey, the MSQOL-54 includes 54 items covering domains such as physical function, cognitive function, emotional well-being, and overall quality of life. This instrument provides a more detailed assessment of the unique challenges faced by PwMS.

### Statistical analysis

All data analyses were performed using IBM SPSS Statistics, Version 29. Descriptive statistics were used to summarize the sample’s demographic and clinical characteristics. Continuous variables were reported as means and standard deviations (SD), while categorical variables were presented as frequencies and percentages.

A series of psychometric analyses were conducted in line with COSMIN guidelines to evaluate the measurement properties of the EQ-5D-5 L in Italian PwMS. These included assessments of test-retest reliability, construct validity (via hypothesis testing for correlations), and known-groups validity.

The present study focused on selected measurement properties, whereas content validity and other COSMIN-defined properties were not directly assessed. Indices related to interpretability, such as floor and ceiling effects, were not included in the predefined analyses.

#### Test-retest reliability

Test-retest reliability was evaluated by administering the EQ-5D-5 L to a subsample of clinically stable participants after a 48-hour interval. Clinical stability was defined as absence of relapse, acute worsening, or any change in disease-modifying treatment during the interval. The Intraclass Correlation Coefficient (ICC) was calculated using a two-way random-effects model with absolute agreement. ICC values of ≥ 0.70 were interpreted as acceptable indicators of reproducibility over time.

#### Construct validity

Construct validity was assessed through hypothesis testing for correlations, in accordance with COSMIN guidelines. Specifically, concurrent validity was evaluated by examining the association between the EQ-5D-5 L and the MSQOL-54, a disease-specific HRQoL instrument designed for PwMS. Pearson’s correlation coefficients were calculated between the EQ-5D-5 L index and Visual Analogue Scale (VAS) scores and corresponding domains of the MSQOL-54.

Prior to analysis, a set of a priori hypotheses was formulated to guide interpretation of the results. These hypotheses predicted strong positive correlations (*r* ≥ 0.60) between conceptually related domains, such as:

- the EQ-Index score and the MSQOL-54 physical function domain,
- the EQ-Index score and the Physical Health Composite Score (PH_CS) of the MSQOL-54,
- the EQ-VAS score and the Overall Quality of Life (Overall_QoL) item of the MSQOL-54.

We expected moderate correlations (0.30 ≤ *r* < 0.60) between less directly related but conceptually overlapping dimensions, and weak or no correlation (*r* < 0.30) with unrelated domains such as role limitations due to emotional problems. The direction and magnitude of these correlations were hypothesized based on theoretical relationships and evidence from previous validation studies of the EQ-5D-5 L in neurological populations [23, 30].

Although directional hypotheses were defined a priori, two-tailed testing was employed to remain conservative in estimating statistical significance and to account for the possibility of unexpected inverse associations in complex multidimensional constructs such as HRQoL.

#### Known-groups validity

Known-groups validity was assessed to determine the instrument’s ability to differentiate between subgroups with theoretically different health statuses, rather than its cross-cultural comparability. Specifically, one-way ANOVA was used to compare EQ-Index and EQ-VAS scores across disability groups, as defined by EDSS categories (mild, moderate, severe). Additionally, independent samples Student T-tests were employed to assess differences based on gender, employment status, and engagement in physiotherapy.

These subgroup analyses were performed to examine the influence of demographic and clinical factors on the EQ-5D-5 L index and VAS scores.

## Results

### Sample characteristics

A total of 69 participants diagnosed with MS were included in the study, comprising 38 females (55%) and 31 males (45%), with a mean age of 49 ± 13 years. Although the sample size was limited, the sample included participants with heterogeneous sociodemographic and clinical characteristics, allowing evaluation of the measurement properties of the instrument across different clinical profiles. No data were missing or excluded during analysis, and all participants completed the full set of questionnaires. No missing responses were observed for the EQ-5D-5 L or EQ-VAS. Descriptive statistics of EQ-5D-5 L Index and EQ-VAS scores are reported to describe the distribution of scores in the study sample. There were no dropouts or incomplete records.

Specifically, 73.9% of participants had completed at least a high school education, 52.2% were employed, and an equal proportion reported undergoing physiotherapy. With respect to disability, 58% of the sample fell within the mild EDSS range (0–3.5), while 10.1% had severe disability (EDSS 6.5–9). The duration since diagnosis was highly variable: 39.1% had been diagnosed within the past 10 years, whereas 17.4% had lived with MS for more than 30 years. In terms of body weight status, 55.1% of participants had a normal BMI, 39.1% were overweight, and 5.8% were underweight. Information on MS clinical phenotype was not consistently available in the clinical records and was therefore not included in the present analyses.

Detailed descriptive statistics are presented in Table 1.

**Table 1:** Sociodemographic and clinical characteristics of the sample (PwMS)

**Age (years), mean ± SD**	49 ± 13.0
**Gender,** ***n*** (%)	
Female	38 (55)
Male	31 (45)
**Education Level**,** n(%)**	
Elementary School	1 (1.4)
Middle School	14 (20.3)
High School Diploma	25 (36.2)
Bachelor’s Degree	10 (14.5)
Master’s Degree	16 (23.2)
PhD	3 (4.3)
**Physiotherapy**,** n (%)**	
Yes	36 (52.2)
No	33 (47.8)
**Employment Status**,** n (%)**	
Employed	36 (52.2)
Unemployed	33 (47.8)
**EDSS**,** n (%)**	
0-3.5 (Mild Disability)	40 (58)
4–6 (Moderate Disability)	22 (31.9)
6.5-9 (Severe Disability)	7 (10.1)
**Time Since Diagnosis (years)**,** n(%)**	
< 10 years	27 (39.1)
10–14 years	5 (7.2)
15–19 years	10 (14.5)
20–29 years	15 (21.7)
> 30 years	12 (17.4)
**BMI**,** n (%)**	
Underweight	4 (5.8)
Normal Weight	38 (55.1)
Overweight	27 (39.1)

BMI = Body Mass Index EDSS = Expanded Disability Status Scale; N = Number of Participants; SD = Standard Deviation

### Psychometric properties of the EQ-5D-5 L

To assess the measurement properties of the EQ-5D-5 L, analyses were conducted to examine test-retest reliability, construct validity (via hypothesis testing), and known-groups validity.

#### Test-retest reliability

Test-retest reliability was evaluated in a subsample of 30 clinically stable participants who completed the EQ-5D-5 L twice over a 48-hour interval. The mean age of the subsample was 43.3 ± 12.1 years, with a balanced gender distribution (50% female). Most participants (80%) had completed at least high school, 60% were employed, and 53.3% reported undergoing physiotherapy. With respect to BMI classification, 60% were normal weight and 40% were overweight. All participants had mild disability (EDSS 0–3.5), as this subgroup was selected to ensure clinical stability during the retest interval; therefore, reliability estimates should be interpreted with caution when extended to individuals with moderate or severe disability.

Intraclass Correlation Coefficients (ICCs) for all five dimensions, the EQ-5D-5 L Index, and the VAS exceeded the conventional threshold of 0.70, indicating good reliability. The highest reliability was observed for the EQ-5D-5 L Index (ICC = 0.945; 95% CI: 0.884–0.974. Slightly lower ICC values were observed for the pain/discomfort and self-care dimensions, although all coefficients remained above the predefined threshold for acceptable reliability, as shown in Table 2.

**Table 2:** Test-retest analysis

EQ-5D-5 L	*N*	Pre-test Mean ± SD	Post-test Mean ± SD	ICC	95% CI (Lower-Upper)
Item 1 (Mobility)	30	2.23 ± 1.14	2.30 ± 1.26	0.910	0.810–0.957
Item 2 (Self-Care)	30	1.53 ± 0.94	1.67 ± 1.21	0.813	0.607–0.911
Item 3 (Usual Activities)	30	2.00 ± 1.11	2.07 ± 1.14	0.913	0.816–0.958
Item 4 (Pain/Discomfort)	30	2.23 ± 1.14	2.23 ± 0.90	0.848	0.681–0.928
Item 5 (Anxiety/Depression)	30	2.07 ± 0.94	2.00 ± 0.95	0.895	0.779–0.950
EQ-Index Score	30	0.72 ± 0.28	0.69 ± 0.35	0.945	0.884–0.974
EQ-VAS Score	30	65.83 ± 21.05	67.57 ± 20.06	0.883	0.754–0.944

CI = Confidence Interval; EQ = Euro QoL; N = Number of Participants; SD = Standard Deviation; VAS = Visual Analogue Scale

#### Construct validity

Construct validity was examined through hypothesis-driven correlation analysis between the EQ-5D-5 L and the MSQOL-54. A set of a priori hypotheses was defined to anticipate strong positive correlations (*r* ≥ 0.60) between conceptually related dimensions, particularly between the EQ-Index and the PH_CS and between the EQ-VAS and the Overall_QoL domain of the MSQOL-54. These findings provided partial evidence of construct validity, consistent with the predefined hypotheses, with strong correlations found between the EQ-Index and the PH_CS (*r =* 0.759, *p <* 0.001) and between the EQ-VAS and the Overall_QoL (*r =* 0.707, *p <* 0.001).

Moderate correlations (0.30 ≤ *r* < 0.60), all statistically significant, were observed between the EQ-5D-5 L scores and several other domains, in line with theoretical expectations of partially overlapping constructs. Specifically, they were found with physical activity (EQ-VAS: *r =* 0.576, *p <* 0.001), energy (EQ-VAS: *r =* 0.513, *p <* 0.001; EQ-Index: *r =* 0.404, *p =* 0.001),* pain* (EQ-VAS: *r =* 0.440, *p <* 0.001; EQ-Index: *r =* 0.416, *p <* 0.001), and social functioning (EQ-VAS: *r =* 0.400, *p =* 0.001; EQ-Index: *r =* 0.464, *p <* 0.001). Correlations with the cognitive functioning domain were modest, suggesting only partial overlap between the constructs measured by the two instruments.

Conversely, weak and mostly non-significant correlations (*r* < 0.30) were observed with domains theoretically unrelated to the EQ-5D-5 L, such as Role Limitation due to Emotional Problems, which showed negligible and non-significant associations with both the EQ-VAS (*r* = 0.228, *p* = 0.059) and the EQ-Index (*r* = 0.052, *p* = 0.672). These findings further support the discriminant validity of the instrument. The detailed results of these correlations are presented in Table 3.

**Table 3:** Pearson’s correlation coefficient between EQ-Index Score, EQ-VAS Score, and MSQOL-54 Domains

MSQOL-54 domains		EQ-VAS score		EQ-Index score			
	*N*	Pearson’s *r*	*P*-value	Pearson’s *r*	*P*-value		
Physical Activity	69	0.576	< 0.001	0.759	< 0.001		
Role Limitation due to Physical Problems	69	0.435	< 0.001	0.286	0.017		
Role Limitation due to Emotional Problems	69	0.228	0.059	0.052	0.672		
Pain	69	0.440	< 0.001	0.416	< 0.001		
Emotional Well-Being	69	0.272	0.024	0.327	0.006		
Energy	69	0.513	< 0.001	0.404	0.001		
Health Perception	69	0.488	< 0.001	0.404	0.001		
Social Functioning	69	0.400	0.001	0.464	< 0.001		
Cognitive Functioning	69	0.393	0.001	0.249	0.039		
Health Distress	69	0.417	< 0.001	0.461	< 0.001		
Sexual Functioning	69	0.268	0.026	0.350	0.003		
Change in Health	69	0.480	< 0.001	0.276	0.022		
Satisfaction with Sexual Functioning	69	0.313	0.009	0.365	0.002		
Overall Quality of Life	69	0.707	< 0.001	0.663	< 0.001		
Physical Health Composite Score	69	0.639	< 0.001	0.642	< 0.001		
Mental Health Composite Score	69	0.474	< 0.001	0.353	0.003		

EQ = Euro QoL; N = Number of Participants; MSQOL = Multiple Sclerosis Quality of Life

#### Known-groups validity

Differences in EQ-5D-5 L scores were examined across various demographic and clinical subgroups to assess the instrument’s ability to differentiate between distinct population characteristics. One-way ANOVA revealed statistically significant differences in both EQ-Index and EQ-VAS scores across EDSS-defined disability categories. Participants with mild disability (EDSS 0–3.5) reported significantly higher mean scores than those with moderate (EDSS 4–6) or severe disability (EDSS 6.5–9), with *p* < 0.001 for both comparisons.

Further analyses examined the impact of employment status and participation in physiotherapy programs on quality of life outcomes. Employed participants demonstrated significantly higher EQ-Index scores compared to their unemployed counterparts (*p* = 0.001).

Participants engaged in physiotherapy programs reported lower EQ-Index scores than those not involved in rehabilitation activities (*p* < 0.001); however, this comparison should be interpreted descriptively, as participation in rehabilitation may reflect greater clinical severity rather than a direct influence on quality of life. Comparisons involving education level and BMI should be considered exploratory, as some categories included a limited number of participants.

The detailed results of these subgroup analyses are presented in Table 4, highlighting the instrument’s ability to capture variations in health-related quality of life across different people groups.

**Table 4:** Comparison of EQ-5D-5 L Scores Across Sociodemographic and Clinical Subgroups

	*N* (%)	EQ-Index score (Mean ± SD)	*P*-value	EQ-VAS score (Mean ± SD)	*P*-value
**Gender**					
Female	38 (55)	0.74 (± 0.26)	0.237	63.55 (± 19.34)	0.340
Male	31 (45)	0.61 (± 0.30)		60.65 (± 23.41)	
**Education Level**					
Elementary School	1 (1.4)	0.48	0.339	40.0	0.695
Middle School	14 (20.3)	0.59 (± 0.30)		57.86 (± 16.38)	
High School	25 (36.2)	0.73 (± 0.29)		65.00 (± 22.31)	
Bachelor’s Degree	10 (14.5)	0.59 (± 0.38)		58.50 (± 24.61)	
Master’s Degree	16 (23.2)	0.77 (± 0.16)		66.25 (± 21.49)	
PhD	3 (4.3)	0.61 (± 0.37)		58.33 (± 24.66)	
**Physiotherapy**					
Yes	36 (52.2)	0.56 (± 0.33)	< 0.001	56.11 (± 22.96)	0.097
No	33 (47.8)	0.81 (± 0.15)		68.94 (± 16.90)	
**Employment Status**					
Employed	36 (52.2)	0.79 (± 0.20)	0.001	69.31 (± 19.28)	0.341
Unemployed	33 (47.8)	0.56 (± 0.32)		54.55 (± 20.67)	
**EDSS**					
0-3.5 (Mild)	40 (58)	0.85 (± 0.10)	< 0.001	72.86 (± 14.77)	< 0.001
4–6 (Moderate)	22 (31.9)	0.62 (± 0.26)		51.84 (± 22.56)	
6.5-9 (Severe)	7 (10.1)	0.37 (± 0.24)		59.17 (± 14.97)	
**Time Since Diagnosis**					
< 10 years	27 (39.1)	0.70 (± 0.32)	0.953	65.00 (± 21.35)	0.234
10–14 years	5 (7.2)	0.71 (± 0.16)		68.00 (± 22.80)	
15–19 years	10 (14.5)	0.72 (± 0.28)		53.50 (± 25.39)	
20–29 years	15 (21.7)	0.64 (± 0.32)		68.00 (± 17.91)	
> 30 years	12 (17.4)	0.66 (± 0.22)		53.75 (± 18.36)	
**BMI**					
Underweight	4 (5.8)	0.62 (± 0.23)	0.912	47.50 (± 28.72)	0.215
Normal Weight	38 (55.1)	0.69 (± 0.28)		65.39 (± 21.70)	
Overweight	27 (39)	0.68 (± 0.30)		60.00 (± 18.76)	

BMI =Body Mass Index; EDSS = Expanded Disability Status Scale; EQ = Euro QoL; N = Number of Participants; SD = Standard deviation

## Discussion

This study aimed to assess the psychometric properties of the Italian version of the EQ-5D-5 L in people with Multiple Sclerosis (PwMS). The findings demonstrate that the instrument shows satisfactory measurement properties in this population. In particular, the EQ-5D-5 L showed high test-retest reliability, and partial evidence of construct and known-groups validity. These results support its utility as a generic HRQoL instrument, while also underscoring important limitations in fully capturing MS-specific health domains [31].

Test-retest reliability was excellent, with ICCs ranging from 0.813 to 0.945. The EQ-Index score achieved the highest ICC, indicating high temporal stability in clinically stable participants. These findings support the reproducibility of the EQ-5D-5 L over short intervals in clinically stable participants, aligning with psychometric data from prior studies in other chronic neurological diseases such as Alzheimer’s and epilepsy [32–34]. Notably, the use of a short 48-hour retest interval minimized the likelihood of health state change, allowing for a more accurate estimate of measurement stability. It is also important to note that test–retest reliability was assessed exclusively in participants with mild disability (EDSS ≤ 3.5), who were selected to ensure clinical stability during the retest interval. This methodological decision aimed to minimize day-to-day symptom fluctuations—such as fatigue or pain—more commonly observed in individuals with higher disability levels, which could potentially affect reproducibility estimates. However, this approach inherently limits the generalizability of test–retest results to the broader MS population, particularly those with moderate to severe functional impairment.

Construct validity was assessed through hypothesis-driven correlation analyses with the MSQOL-54. As expected, strong correlations emerged between the EQ-Index and the PH_CS (*r* = 0.759), and between the EQ-VAS and the Overall_QoL item (*r* = 0.707), providing partial evidence of concurrent validity. However, demonstrating construct validity involves more than the identification of statistically significant correlations; it requires a clear theoretical rationale supporting the expected relationships. In this study, such justification was ensured through a priori hypotheses grounded in the conceptual overlap between the two instruments. Similar validation strategies have been employed in previous study of the EQ-5D-5 L in PwMS conducted in other linguistic and cultural contexts, including German sample, which reported comparable correlation patterns between EQ-5D scores and disease-specific measures [35].

Nonetheless, content validity was not formally assessed in the present study. According to COSMIN standards, content validity represents a fundamental measurement property and should ideally be evaluated through qualitative methods and expert or patient input. Therefore, the considerations reported below should be interpreted as conceptual reflections on the known limitations of generic instruments, rather than as empirical evidence derived from the present analyses. As a generic measure, the EQ-5D-5 L may not fully capture domains that are particularly relevant in MS, such as fatigue, cognitive dysfunction, and social participation [36]. These aspects are frequently underrepresented in generic HRQoL tools, as highlighted in recent studies advocating the inclusion of bolt-on items to enhance sensitivity to disease-specific impacts. Although fatigue was not directly assessed in our sample, it is widely acknowledged as a prevalent and disabling symptom among PwMS, frequently impacting domains such as usual activities and pain/discomfort. Nonetheless, the EQ-5D-5 L lacks a dedicated item to specifically capture fatigue. Therefore, despite the well-documented impact of fatigue on HRQoL in MS, the instrument does not permit an isolated analysis of its contribution.

Similarly, while cognitive dysfunction is widely recognized as a major determinant of QoL in MS, our study did not incorporate direct measures of cognitive performance nor stratify findings by cognitive status. As such, although the importance of cognitive aspects is acknowledged, our data do not allow for inferences regarding the EQ-5D-5 L’s ability to capture this domain. This limitation underscores the need for future research to examine the influence of cognitive function on EQ-5D-5 L scores and to evaluate the potential utility of cognition-specific bolt-on items, as explored in studies involving Amyotrophic Lateral Sclerosis (ALS) and other progressive neurological conditions [37–39].

Known-groups validity was confirmed by the ability of the EQ-5D-5 L to differentiate among people with varying levels of disability, as measured by the EDSS. Significant differences in both EQ-Index and EQ-VAS scores were observed across EDSS-defined groups, with lower HRQoL scores reported by participants with greater disability. This pattern mirrors findings from international studies conducted in Australia, Lebanon, and Germany, suggesting that the EQ-5D-5 L is sensitive to functional impairment even across diverse cultural settings [6, 40, 41]. However, this observation alone does not support claims about cross-cultural validity. Rather, cross-cultural validity refers to the instrument’s equivalence across linguistic and cultural groups and is best assessed through techniques such as differential item functioning (DIF) or multi-group confirmatory factor analysis—methods that were beyond the scope of this study [42].

Interestingly, no statistically significant differences in HRQoL were observed across time-since-diagnosis groups. Although previous studies have reported a decline in quality of life with increasing disease duration and age, our findings may reflect the high interindividual variability typical of MS progression [43, 44]. Indeed, time since diagnosis does not always correspond to disease severity or current functional status, and is therefore a less reliable indicator of HRQoL than metrics such as the EDSS [45]. Moreover, the use of a generic instrument like the EQ-5D-5 L—rather than a disease-specific tool—may have reduced sensitivity to differences related to chronicity. Additionally, our choice to categorize time since diagnosis, combined with the limited sample size in some strata, may have contributed to the absence of detectable differences. This finding may reflect the heterogeneous clinical course of MS and the limited ability of temporal indicators alone to capture variations in perceived health status [46].

Further subgroup analyses showed that employed participants and those not undergoing physiotherapy reported higher HRQoL scores, likely reflecting better overall functional status. These findings are consistent with prior literature, where employment has been associated with enhanced perceived health, social integration, and economic stability in PwMS [47]. Likewise, not being enrolled in rehabilitation services may reflect lower disease burden rather than inferior access to care [36].

Another limitation of this study lies in the relatively modest sample size, which may affect the generalizability and precision of the findings. Nonetheless, the use of Classical Test Theory methods—such as test–retest reliability, and construct validity—was appropriate for the study aims and sample size. Recruitment was conducted in a real-world clinical setting using strict eligibility criteria to ensure clinical stability between assessments. While this approach enhances the internal validity of test–retest reliability estimates, it also constrains recruitment capacity, especially in a population characterized by chronicity and clinical heterogeneity such as MS.

Moreover, the test–retest reliability analysis was conducted exclusively on participants with mild disability (EDSS ≤ 3.5), a methodologically sound decision aimed at ensuring clinical stability during the retest interval. However, this selective sampling introduces a potential bias that limits the external validity of reproducibility estimates. The resulting homogeneity of the test–retest subgroup precludes evaluation of the instrument’s temporal stability across the full spectrum of disease severity, particularly in individuals with moderate to severe disability, who often exhibit greater symptom fluctuation.

Additionally, data regarding the distribution of MS phenotypes (i.e., RRMS, SPMS, PPMS) were not available for the present sample due to limitations in the clinical records retrieved at the time of data collection. While this precluded stratified analyses by disease course, it reflects a common constraint in real-world observational studies conducted outside of dedicated MS registries and further underscores the need for standardized clinical documentation in routine care settings.

Future studies are warranted to replicate and extend these findings in larger and more heterogeneous cohorts, encompassing a broader range of functional status in PwMS.

In conclusion, the Italian version of the EQ-5D-5 L demonstrated satisfactory measurement properties in PwMS and can be considered an appropriate tool for assessing generic HRQoL in this population. Importantly, the instrument demonstrated satisfactory measurement properties in this sample, although test–retest reliability was evaluated only in participants with mild disability, and therefore the generalizability of reproducibility estimates to individuals with moderate or severe impairment should be interpreted with caution. Nevertheless, its limitations in content coverage, particularly regarding fatigue and cognitive function, suggest that it should be used in conjunction with disease-specific instruments when comprehensive assessment is required. Future research should investigate the feasibility and added value of bolt-on items targeting MS-relevant domains, as well as explore the cross-cultural validity of the EQ-5D-5 L using advanced psychometric methods.

## Conclusion

This study provides a psychometric evaluation of selected measurement properties of the Italian EQ-5D-5 L in people with Multiple Sclerosis (PwMS), demonstrating satisfactory measurement properties in terms of test-retest reliability, construct validity, and known-groups validity. The results support the use of the EQ-5D-5 L as a generic instrument for assessing in Italian PwMS within both clinical and research contexts.

The instrument effectively distinguished between groups stratified by disability severity, employment status, and engagement in physiotherapy, highlighting its ability to detect meaningful variations in self-perceived health status. These findings are consistent with international literature and confirm the EQ-5D-5 L’s utility in capturing functional heterogeneity in chronic neurological populations.

Therefore, although the EQ-5D-5 L represents a reliable and efficient tool for monitoring general HRQoL trends, it should be systematically complemented by disease-specific instruments to achieve a comprehensive understanding of PwMS experiences.

Nonetheless, the interpretation of our findings should take into account certain methodological limitations, particularly the relatively small sample size and the homogeneity of the test–retest subgroup, which included only participants with mild disability. These factors may restrict the generalizability of reliability estimates to the wider MS population.

This dual approach enhances the clinical utility of PROMs, enabling more informed decision-making in personalized care and supporting health policy strategies aimed at optimizing resource allocation and service design.

Future studies should explore the integration of MS-relevant bolt-on items and examine cross-cultural validity using advanced psychometric methods such as differential item functioning and multi-group analyses.

In conclusion, this validation represents an important contribution toward the culturally sensitive application of PROMs in Italian PwMS and supports the inclusion of the EQ-5D-5 L in outcome assessment strategies aimed at enhancing person-centered care, guiding clinical decisions, and informing health policy in the management of chronic neurological diseases.

## Data Availability

Available from the corresponding author on reasonable request.

## Abbreviations

ANOVA: Analysis of Variance
CI: Confidence Interval
COSMIN: COnsensus–based Standards for the selection of health Measurement INstruments
EDSS: Expanded Disability Status Scale
EQ: 5D–5 L–EuroQol five–dimension five–level questionnaire
EQ: VAS–EuroQol Visual Analogue Scale
FAMS: Functional Assessment of Multiple Sclerosis
HRQoL: Health–Related Quality of Life
ICC: Intraclass Correlation Coefficient
MS: Multiple Sclerosis
MSIS: 29–Multiple Sclerosis Impact Scale
MSQOL: 54–Multiple Sclerosis Quality of Life–54
MusiQoL: Multiple Sclerosis International Quality of Life questionnaire
PROM: Patient–Reported Outcome Measure
PwMS: People with Multiple Sclerosis
SD: Standard Deviation
